# Development of Thermoplastic Starch (TPS) Including Leather Waste Fragments

**DOI:** 10.3390/polym12081811

**Published:** 2020-08-12

**Authors:** Silvio Pompei, Jacopo Tirillò, Fabrizio Sarasini, Carlo Santulli

**Affiliations:** 1 School of Architecture and Design, Università di Camerino, viale della Rimembranza, 63100 Ascoli Piceno, Italy; silvio.pompei@studenti.unicam.it; 2 Department of Chemical Engineering Materials Environment and UdR INSTM, Sapienza Università di Roma, Via Eudossiana 18, 00184 Roma, Italy; jacopo.tirillo@uniroma1.it (J.T.); fabrizio.sarasini@uniroma1.it (F.S.); 3 School of Sciences and Technologies, Università di Camerino, via Gentile III da Varano 7, 62032 Camerino, Italy

**Keywords:** leather waste, thermoplastic starch, mechanical characterization, thermal characterization

## Abstract

A thermoplastic starch (TPS) material is developed, based on corn starch plasticized with glycerol and citric acid in a 9:3:1 ratio and further bonded with isinglass and mono- and diglycerides of fatty acids (E471). In TPS, leather fragments, in the amount of 7.5 15 or 22.5 g/100 g of dry matter, were also introduced. The mixture was heated at a maximum temperature of 80 °C, then cast in an open mold to obtain films with thickness in the range 300 ± 50 microns. The leather fragments used were based on collagen obtained from production waste from shoemaking and tanned with tannins obtained from smoketree (*Rhus cotinus*), therefore free from chromium. Thermogravimetric (TGA) tests suggested that material degradation started at a temperature around 285 °C, revealing that the presence of leather fragments did not influence the occurrence of this process in TPS. Tensile tests indicated an increase in tensile properties (strength and Young’s modulus) with increasing leather content, albeit coupled, especially at 22.5 wt%, with a more pronounced brittle behavior. Leather waste provided a sound interface with the bulk of the composite, as observed under scanning electron microscopy. The production process indicated a very limited degradation of the material after exposure to UV radiation for eight days, as demonstrated by the slight attenuation of amide I (collagen) and polysaccharide FTIR peaks. Reheating at 80 °C resulted in a weight loss not exceeding 3%.

## 1. Introduction

Leather is obtained from animal hides, which are based on collagen, a protein molecule constituted by sequential chains of amino acids, twisted and bound in a strong molecular structure made of fibers. The process of leather fabrication does require its conservation for manipulation through tanning, which involves the fixation of tannins to the collagen matrix. The effect of tannins is enabling preservation, making leather imputrescible, therefore treatable with chemicals, while improving its hardness and strength. If trivalent chromium salts are used for this purpose, leather waste is classified as a special refuse, which requires specific methods for handling to prevent chromium from penetrating into the soil or underground water [1]. To improve leather sustainability and ease its disposal, vegetable tannins can be employed, such as those obtained from smoketree (*Rhus cotinus*). This was a traditional use in some regions, such as Marche and Tuscany, of Central Italy, and has currently a reprise, other than on sustainability grounds, also in connection with the revived use of natural colors, of some of which tannin is an essential element [2]. In this way, the structural and functional advantages of chromium tanning are preserved, while, on the other side, leather waste can be disposed of e.g., in other biodegradable materials [3]. The waste from leather treated with vegetable tanning proved to be microporous and functional for sustainable use, as it was proposed after chemical activation with alkali, for the removal of volatile organic compounds (VOCs), such as toluene [4]. 

Starting from these considerations, the waste from vegetable-tanned leather could represent a candidate for possible inclusion into biopolymers such as thermoplastic starches (TPS) based on starch-glycerol. This procedure demonstrated to be suitable for the introduction of fillers in powder form, such as clay, even with limited control of their dimensions [5], or garden waste, such as the one from Opuntia, in fibrous form [6]. In general terms, this waste could be introduced in the matrix without any further treatment, although obviously with quite limited performance. As a consequence, these were able to effectively include waste, mainly from the agricultural food and non-food sector [7,8]. The aforementioned limited performance, in the case of film fabrication, can derive also from the difficult control of flowability: in this sense, citric acid, a cheap and easily available chemical, can provide some better compatibility with starch, therefore easing film processing [9]. The limitation of TPS is given by their glass transition temperature T_g_, which is usually around 80 °C, yet strongly dependent on the amount of water added. Above T_g_, thermoplastic starch loses its mechanical properties, and considerably swells in an irreversible way [10]. This phenomenon requires putting some attention into the creation of a suitable mixture for inclusion of other materials, especially when, as it is the case with films, the dimensional tolerance is very limited. The main waste constituting leather fragment is collagen, which has been added with starch in applications linked to food preservation (e.g., sausage casing): the addition of not exceedingly large amounts of starch (less than 50 wt%) to collagen was demonstrated as being effective for enhancing film strength and improving durability [11]. 

This study aims particularly to disclose the possibility of re-using environmentally friendly waste material, such as vegetable-tanned leather, which is currently disposed in general waste, because the limited and local characteristics of the productive system do not suggest transportation for recycling as an economically viable option. The application proposed involves its inclusion in self-developed thermoplastic starch (TPS), with the idea to optimize the composition of TPS, in view of its compatibility with waste, to be used as filler. The biodegradability of the composite obtained allows its possible application in products where contact with soil and progressive non-toxic degradation is required, such as it is the case for the on-site production of mulching films. 

## 2. Materials and Methods 

### 2.1. Material Development

#### 2.1.1. Raw Materials Used

**Figure 1:**
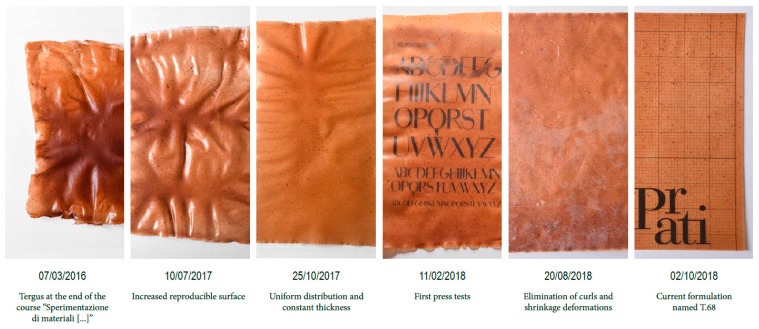
Experimental process leading to the development of the film.

The mixture used for the production of the film included commercial corn starch for alimentary use, glycerol of 99.5% purity (E422), citric acid from lemon juice and isinglass to ease bonding, in the respective proportions 9:3:1:0.7. Moreover, 0.15 parts of mono- and diglycerides of fatty acids (E471) were added to prevent the formation of mold and to ease the manufacturing of a flat film of material during laying up. In this way, a thermoplastic starch (TPS) has been obtained. Smoketree-tanned leather in irregular fragments having their largest dimension between 10 and 500 microns approximately, was added to the material in the proportions of 7.5, 15 and 22.5 wt% over the dry weight of the mixture. The three composites obtained were defined ex-post as TPS_ leather 1/2, TPS_leather 1 and TPS_leather 3/2, considering 1 as the ideal proportion for the production of the final material, which requires some deformation to be retained in the flat sheet obtained. These proportions were set after a large number of experiments carried out starting from October 2015 in the Course of “Sperimentazione di Materiali Innovativi per il Design” (Experimentation on Innovative Materials for Design) in University of Camerino and completed during 2018 with the experimental aims that are illustrated in Figure 1: the last image of Figure 1 shows the material developed, which is here reported as “TPS_Leather 1”, further developed in sheets with dimensions 300 × 200 mm. The contents of the different raw materials in percentage over dry weight of the film are reported in Table 1. The three composites are compared between them, and to the pure TPS developed with the aforementioned proportions.

#### 2.1.2. Film Production 

**Figure 2:**
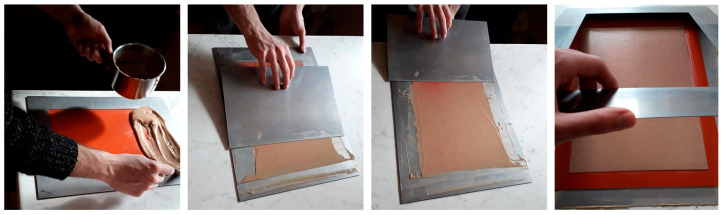
The different phases of the film casting.

Film production was obtained by adding the mixture with some amount of water to allow obtaining a sufficient fluidity, then heating it at a temperature not exceeding 80 °C, reached by its continuous mixing in an uncovered container in 10 min. The mixture was then cast on a silicon plate, 1 mm thick, with the assistance of a steel lamina, again 1 mm thick, to level it inside a steel frame with a thickness of 1.5 mm. The frame is removed just after the cast process and the film is left cooling naturally on the silicon sheet. The phases of the production process are reported in Figure 2. 

**Figure 3:**
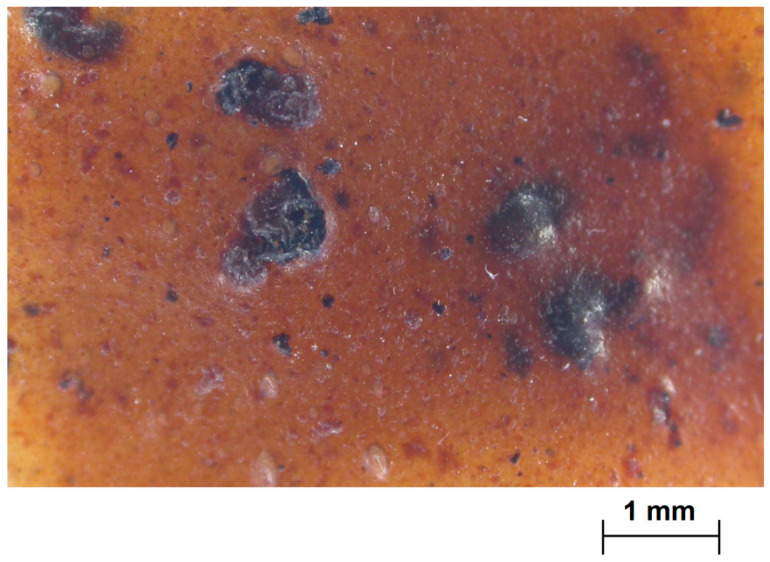
Macrograph of the material with fragments of leather waste.

**Figure 4:**
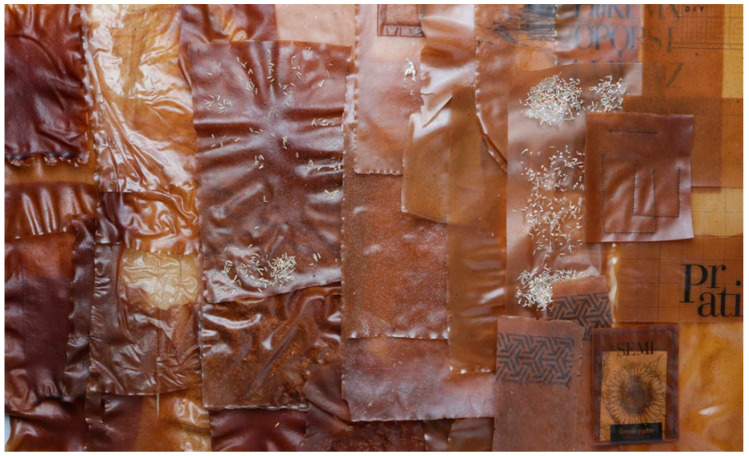
Coupling of the different films strips with lawn seeds prepared for application.

The natural process of film drying, which typically lasted 5 days, made it possible to finally obtain a film of rather constant thickness, in the order of 300 ± 50 microns. From Figure 3, it is possible to observe the interfacial adhesion of leather fragments to the substrate, despite the irregularity of the filler. The films, cut into rectangular strips with maximum dimensions around 150 × 100 mm, are supposed to be coupled in the way reported in Figure 4, therefore partially superimposed to each other. They would serve as the support for texturized lawn modules, able to support small seeds for the growth of plants, therefore, the requirement is more on the effective integration of leather filler than in the creation of larger pieces.

### 2.2. Experimental Methods 

#### 2.2.1. Ageing Tests

Ageing tests were carried out by exposing materials to a gallium-doped Helios (Helios Quartz Group SA, Novazzano, Switzerland) medium pressure mercury lamp, model HMPL, which has emission peaks in the region between 400 and 430 nm, therefore in the IR range, yet it also has considerable emissions in the UV range between 200 and 400 nm. The light exposition was continuative for a period of 192 h. FTIR analysis was carried out using a Perkin Elmer (Milan, Italy) Spectrometer 100 in attenuated total reflection (ATR) mode. A spectral resolution of 3 cm^−1^ in the range (4000–600 cm^−1^) with 512 scans was adopted to record the spectra. Two spectra were carried out, to compare those obtained from new and aged materials.

Tests with Radwag (Radom, Poland) MA 110.R thermobalance involved a program of heating with 5 min at 40 °C, 5 min at 60 °C and 8 min at 80 °C, therefore for a total duration of 18 min. Heating was applied on square samples of 20 mm side, removed by cutting from the rectangles of materials, on which mass was measured every 10 s with an accuracy of ±1 mg. 

#### 2.2.2. Mechanical Characterization of Films 

Specimens for the mechanical characterization were cut from the films in accordance with UNI EN ISO 527-2 (Type 1BA samples with a gauge length of 30 mm). Tensile tests were performed at room temperature in displacement control with a crosshead speed of 2.5 mm/min by using a Zwick/Roell Z010 (Ulm, Germany) universal testing machine. The results are the average of five tests.

#### 2.2.3. Thermal Characterization of Films 

The thermal stability of the films was investigated by thermogravimetric analysis (TGA). To this purpose, a SETSYS Evolution system by Setaram (Caluire, France) was used, heating the samples from 25 °C to 800 °C, with a heating rate of 10 °C/min in a nitrogen atmosphere.

#### 2.2.4. Morphological Characterization by SEM 

The fracture morphology of samples failed in tension was investigated by field emission scanning electron microscopy (Mira3 by Tescan, Brno, Czech Republic). Specimens were sputter coated with gold prior to analysis.

## 3. Results and Discussion

The tensile test results, reported in Table 2, indicated in general a limited variation in terms of stress, strain and stiffness, with respect to other typical materials including organic waste. A comparison was also possible with similar thermoplastic starches with no fillers e.g., those examined in [12]. This reference suggests that the plasticization effect is in the present study much more contained, as observed by the elongation at break for the pure TPS, which just exceeds 50%, making the material quite controllable and with predictable properties during use. In particular, a considerably lower deformation was measured with respect to what was observed in other studies, such as [13], on glycerol and citric acid on corn starch tensile properties.

**Figure 5:**
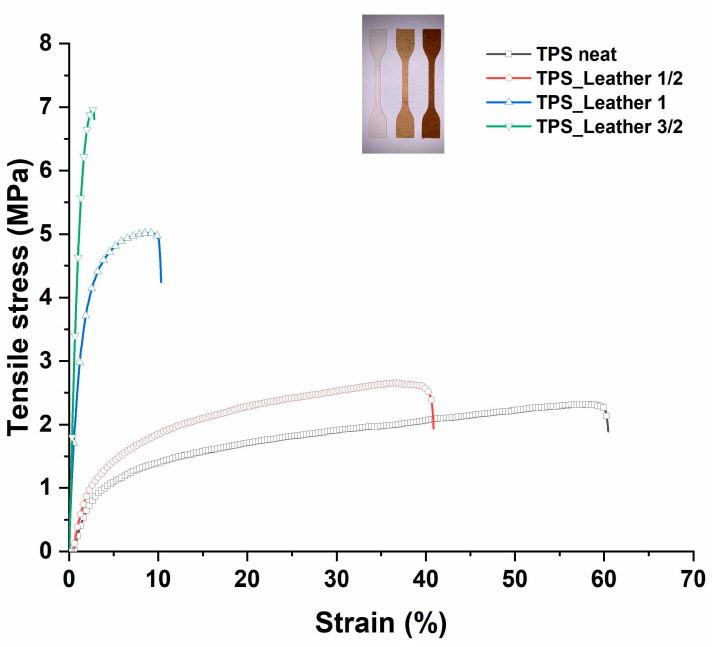
Typical tensile stress-strain curves.

The mechanical properties of the developed films compare quite favorably with those reported in other studies, especially for what concerns the Young’s modulus. In particular, in [14], for materials based on cornstarch and glycerol/water as plasticizer reinforced with bacterial and vegetable cellulose (1% and 5% *w*/*w*), a strength in the range 0.5–3.5 MPa and a Young’s modulus in the range 1–20 MPa was reported, while in [15,16], for thermoplastic starch/clay nanocomposites, strength spanned the range from 2 to 28.1 MPa and the modulus from 7.5 to 196 MPa. As far as the introduction of leather fragments is concerned, the effect of 7.5 wt% over 100 parts of TPS (TPS_Leather 1/2) appears limited on the modification of TPS properties, whereas the material results considerably stronger and stiffer when higher amounts of leather fragments are used. However, as inferred from the typical tensile curves in Figure 5, the introduction of 22.5 wt% of leather fragments resulted in a material failing basically with no plastic behavior, which limits its applications, therefore the intermediate solution of 15 wt% of leather fragments appeared the most suitable for the use proposed.

**Figure 6:**
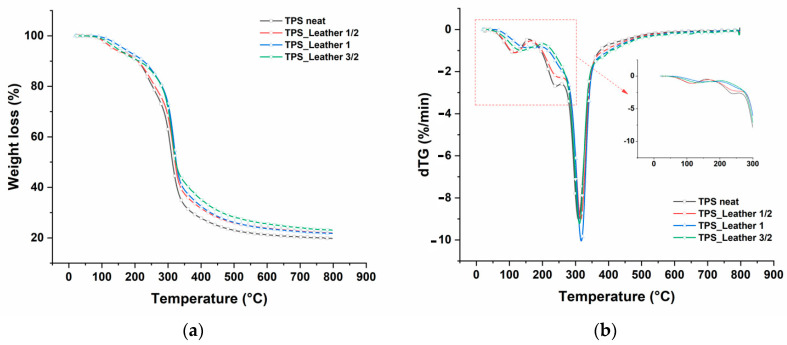
Thermogravimetric curve (TGA) (**a**) and first derivative of the thermogravimetric curve (DTG) (**b**) of thermoplastic starch (TPS) and the different composite materials.

The onset of material degradation started around 285 °C, as determined from the graphical method suggested in [17], for corn starch-glycerol materials, and indicates a typical value for thermoplastic starches. This can suggest that the effect of leather fragments on material degradation is likely to be quite limited. However, the higher amounts introduced offered some reduction of the degradation rate in the region around 250–300 °C, as observable specifically from the inset in Figure 6b. Studies on collagen indicate water loss taking place up to around 125 °C, while the degradation onset of leather is around 300–320 °C [18]. In this case, it is suggested that water loss occurs seamless with desorption of water as the effect of softening of thermoplastic starch: as a matter of fact, the trend appears to be linear up to over slightly 200 °C. Regarding the residual mass at the end of thermal degradation process, a previous study on acrylonitrile-butadiene-styrene (ABS) resin—leather waste composites suggested that leather powder alone was leaving just below 20% of the initial mass at 800 °C [19]. The data found here are basically in line with this indication, suggesting that the presence of leather increases the amount of material normally left after the degradation of thermoplastic starches obtained using corn starch with similar amounts of glycerol [20]. 

**Figure 7:**
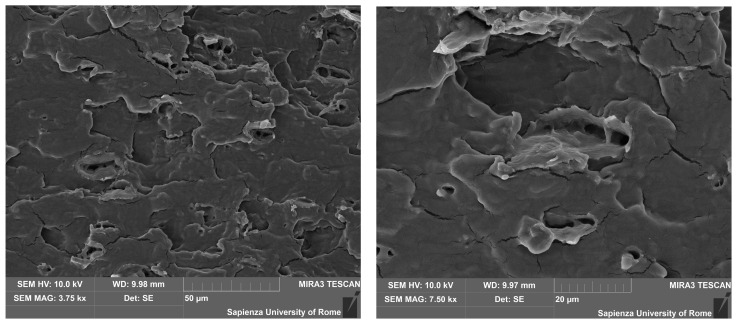
SEM images of the pure TPS fracture surface.

**Figure 8:**
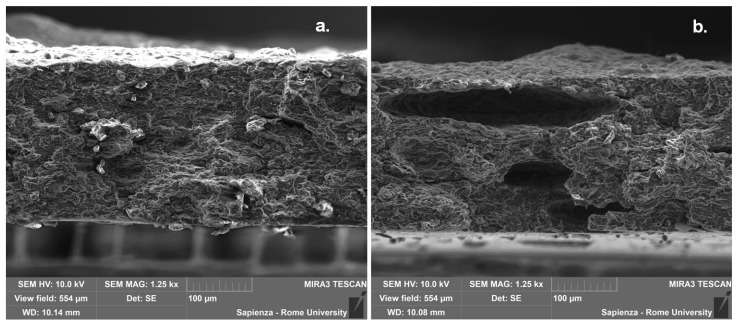
Fracture surface of the composite, without (**a**) and with (**b**) pulled-out leather particles at failure.

**Figure 9:**
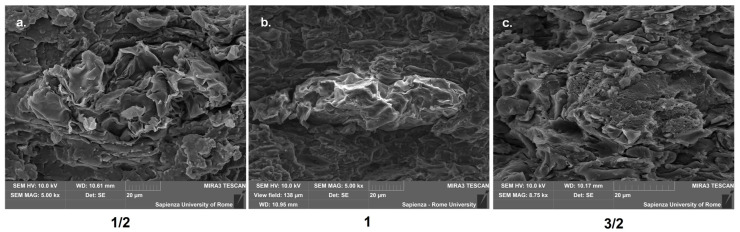
Leather particles embedded in the TPS matrix, 1/2 (**a**), 1 (**b**) 3/2 (**c**).

**Figure 10:**
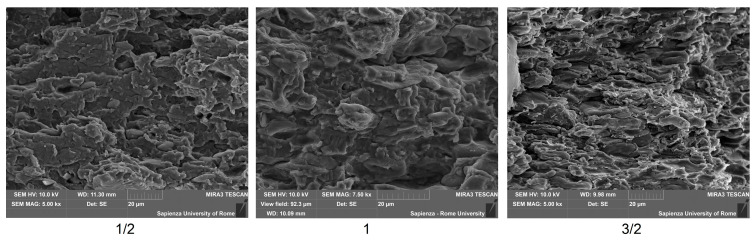
Occurrence of differential deformation in the various composites.

The microscopic characterization of the fracture surfaces indicates for pure TPS a variable fragmentation with loss of material and creation of voids, which is attributable to plasticization effect (Figure 7). In contrast, in the case where leather fragments are introduced, the occurrence of some step-like regions is detected (Figure 8a), which is often encountered in the presence of protein-based structures and likely to be related to the internal layered structure of leather particles [21]. Some pulled-out particles were also observed, as shown in Figure 8b, which suggests the need to improve the interfacial adhesion, because the subsequent detachment of few leather fragments from the matrix would result in the coalescence of voids and sudden failure. On the other side, in most cases the interface proves effective, such as in Figure 9, though its performance might be variable, due to the random distribution of waste particles: in practice, with a lower amount of leather fragments, their boundaries are more recognizable in the TPS matrix (Figure 9a,b), whereas, with their increasing amount, the deformation of the matrix due to the insertion of the filler and the subsequent loading would rather conceal them (Figure 9c). Looking in more detail at the structure of the TPS matrix, some typical situations are recognized during loading, in particular differential deformation, leading in the most critical cases to the widespread formation of cracks (Figure 10). However, it is promising that, in most cases, leather fragments have been shown to provide a strong adhesion to the matrix, although the margins for improvement are surely to be recognized.

In general terms, the material proved suitable for the application envisaged, despite the fact that further mechanical fragmentation of leather waste e.g., by milling, had been purposely avoided. As expected, the dimensional variation of the particles resulted in some further limitation of the plasticization effect provided by the corn starch-glycerol-citric acid interaction, though reduced already by isinglass, as common in TPS [22]. It needs to be considered in any case that the mechanical properties obtained and the thermal stability offered can be considered acceptable: further refinements of the production process and of the film drying uniformity, leading to a stricter dimensional tolerance, would follow in future investigations. 

Regarding the material that proved to be the most suitable for the envisaged application, hence TPS_Leather 1, further characterization was carried out. In particular, a number of peaks were observed from FTIR analysis, whose attribution is reported in Table 3, mainly from the comprehensive and reference study of FTIR spectra on starch, carried out in [23]. Some of these are also corroborated by other information offered in the respective references, and also the peaks referring to the other non-polysaccharide components in the materials, hence glycerol, citric acid and collagen from leather are attributed according to the other references quoted in Table 3. The investigation did not allow the attribution of FTIR peaks to vegetable tannins, despite some studies on this also being available, such as [24], probably due to their amount of leather wastes being too low. 

**Figure 11:**
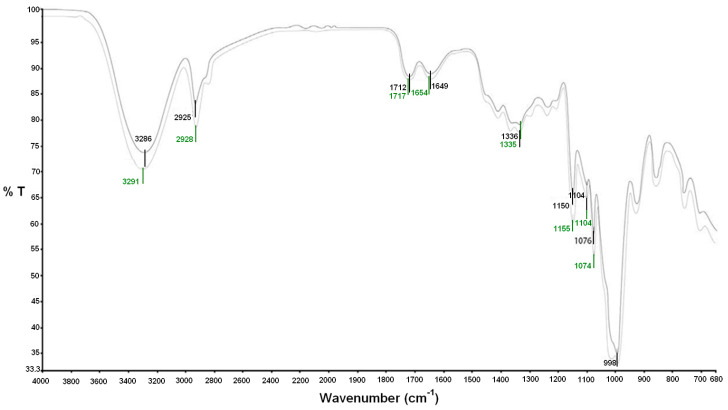
FTIR spectra carried out on the material newly produced (darker curve) and then subjected to UV ageing (lighter curve).

**Figure 12:**
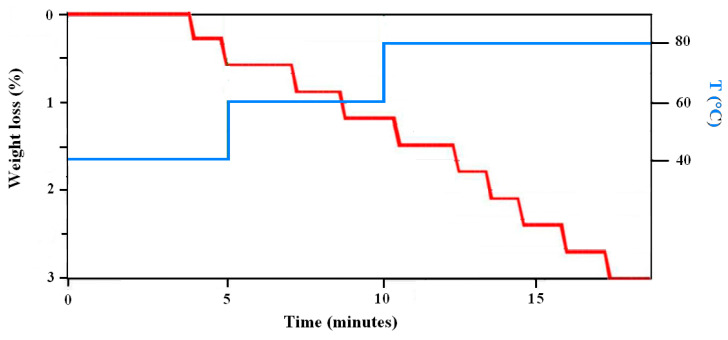
Weight loss of the material during heating up to 80 °C.

Moreover, FTIR analysis suggested that peaks correlated to polysaccharides (starch), to interaction between starch and glycerol, in particular the 1104 cm^−1^, to amide I (collagen) and amino-acid, namely the 998 cm^−1^, only showed, as from Figure 11, a slight degradation after ageing for eight days to simulated exposure to the sunlight. Post-ageing, a re-heating at 80 °C of the material did lead to a water loss not exceeding 3%, as indicated in Figure 12, which demonstrates that most water was linked to the polymeric structure, which could suggest the material is sufficiently strong and stable during storage and application, possibly with limited shrinkage. In fact, coming back to thermogravimetric tests reported in Figure 6, they also suggested a very limited weight loss up to around 80 °C, indicating that the components are hydrated in a stable form. 

## 4. Conclusions

The introduction of leather fragments in three different quantities in a self-produced thermoplastic starch (TPS) based on starch plasticized with glycerol and cross-linked using citric acid proved to be promising. This can be regarded as a sustainable procedure for introducing in soil a biodegradable composite e.g., for lawn growth support, since leather is chrome-free, being smoketree-tanned. The material with intermediate amount of leather fragments did not suffer any significant degradation below 80 °C, which makes it basically acceptable for the application envisaged.

The positive aspects are also in the formation of a sound matrix-filler interface, though with possible improvements based on a more accurate selection of particle distribution. As expected, collagen-based reinforcement does result in hindering the plasticization of TPS, whereas it also provides more controllable mechanical properties. As far as thermal properties are concerned, the introduction of leather fragments does not change to a significant extent the degradation patterns of TPS, which take place just below 300 °C. The amount of leather waste introduced could be possibly increased by working on particle size with more accurate fragmentation methods, while, in the present work, only basic cutting operations have been performed.

## Figures and Tables

**Table 1:** Different components of the film.

Component	Content over Dry Weight (%)
Corn starch	55.2
Glycerol	18.5
Citric acid	6.2
Isinglass	4.3
E471	0.8
Leather fragments	7.5 (leather 1/2), 15 (leather 1) or 22.5 (leather 3/2)

**Table 2:** Summary of tensile test results.

	σ (MPa)	E (MPa)	ε (%)
TPS_neat	2.30 ± 0.02	52.17 ± 7.54	58.60 ± 2.70
TPS_Leather 1/2	2.65 ± 0.09	71.24 ± 6.55	45.30 ± 4.30
TPS_Leather 1	4.75 ± 0.21	200.18 ± 8.04	10.05 ± 2.26
TPS_Leather 3/2	7.27 ± 0.28	577.40 ± 18.79	2.50 ± 0.50

**Table 3:** Attribution of FTIR peaks observed during the scanning.

Peak (cm^−1^)	Attribution
3286–3291	Hydrogen bonds given by hydrolyzing
2925–2928	C–H stretching mode of starch [25,26]
1712–1717	C=O stretching vibration in carboxyl groups due to citric acid [27]
1649–1654	Amide I (collagen) [28]
1335–1336	Polysaccharides C–OH bending [28]
1150–1155	Polysaccharides (starch) C–H bending [29]
1104	C–O stretching vibration peak of glycerol [30]
1074–1076	C–O–H stretching vibration [30]
998	Out-of-plane OH-vibrations in carboxyl (amino-acid) [31]
